# SONIVA database: Speech recognition validation in aphasia

**DOI:** 10.1038/s41597-026-07596-3

**Published:** 2026-06-13

**Authors:** Giulia Sanguedolce, Cathy J. Price, Sophie Brook, Dragos C. Gruia, Niamh V. Parkinson, Patrick A. Naylor, Fatemeh Geranmayeh

**Affiliations:** 1 Department of Computing, Imperial College London, London, UK; 2 Department of Electrical and Electronic Engineering, Imperial College London, London, UK; 3 Department of Brain Sciences, Imperial College London, London, UK; 4 Department of Imaging Neuroscience, University College London, London, UK; 5 Imperial College Healthcare NHS Trust, London, UK

**Keywords:** Diagnostic markers, Translational research, Databases

## Abstract

Post-stroke aphasia is a major contributor to language impairment and neuro-disability worldwide, making automated assessment a critical research priority. However, clinically validated automatic speech recognition (ASR) systems remain limited by the scarcity of large, annotated datasets capturing aphasia’s heterogeneous manifestations. We introduce SONIVA (Speech recOgNItion Validation in Aphasia), the largest and most comprehensively curated database for validating speech recognition in aphasia, comprising audio recordings from approximately 1,000 stroke survivors and 6,000 age-matched controls. The dataset comprises annotated speech from 571 stroke survivors, 103 of whom contributed longitudinal recordings (mean age: 60.65  ± 12.97 years; 68.77% male), and 103 controls (mean age: 59.64  ± 11.48 years; 62.05% male). These recordings are enriched with detailed linguistic coding, orthographic transcriptions, and International Phonetic Alphabet annotations. Foundation models fine-tuned on SONIVA correlate strongly with expert transcriptions (Spearman’s r = 0.79-0.86; *p* < 0.0001), while acoustic classifiers achieve 93% stroke classification accuracy, enabling scalable analysis for rehabilitation and clinical assessment.

## Background

Acquired speech and language disorders pose a significant challenge in neurological diseases, affecting millions of individuals worldwide. These impairments arise from a wide range of conditions, including stroke, traumatic brain injury (TBI), and neurodegenerative diseases such as dementia and Parkinson’s disease^1^. Speech disorders are particularly disabling because they disrupt communication, social interaction, and functional independence, leading to increased healthcare needs, prolonged hospital stays, and higher mortality and morbidity rates. Stroke is one of the leading causes of acquired speech and language disorders, with up to 40% of survivors experiencing communication impairments, ranging from motor speech disorders (dysarthria, apraxia of speech) to linguistic deficits (aphasia). Among these conditions, aphasia, a language disorder affecting speech production, comprehension, reading, and writing, is particularly prevalent in stroke, affecting one-third of survivors, though it also occurs in dementia and TBI^1^. Aphasia alone leads to significant healthcare demands, with longer hospitalisation times and increased reliance on rehabilitation and caregiving services. The economic burden of post-stroke aphasia is estimated at 3.41 billion USD annually, including medical costs, assistive technologies, lost productivity, and caregiver support^2^. Given these substantial clinical and socioeconomic impacts, improving the diagnosis and treatment of aphasia and other communication disorders has been recognised as a critical research priority for patients and stakeholders^3^. A major limitation of current clinical practice is the lack of scalable, objective tools for speech assessment. In this context, artificial intelligence (AI)-powered speech recognition technology presents a promising solution. By leveraging automated speech recognition (ASR) models trained on aphasic speech cohorts, AI can facilitate earlier diagnosis, continuous monitoring, and more accessible rehabilitation, which may support both assessment and long-term management in clinical settings.

Datasets from stroke-induced speech impairments represent a particularly strong candidate for training ASR models, not only due to the high prevalence of aphasia but also because post-stroke deficits exhibit significant heterogeneity both across patients and within the same individual over time^4^. Unlike neurodegenerative diseases, where speech deterioration is progressive and relatively predictable, stroke-related impairments emerge acutely, with highly variable severity and recovery trajectories depending on the location and extent of brain damage. This variability presents both a challenge and an opportunity for developing robust pathological speech models capable of handling diverse speech deficits, applicable to a multitude of neurological disorders^5^.

Stroke survivors typically experience a range of linguistic and motor impairments that collectively compromise communication and daily functioning. Among these, aphasia primarily affects the linguistic system^6^, characterised by phonemic errors (incorrect sounds), semantic paraphasias (meaning-related word substitutions), disrupted speech rate, and various dysfluencies^7^. Patients with aphasia may also exhibit syntactic errors (omitting function words and grammatical morphemes), anomia (word-finding difficulties), and neologisms (invented words)^8^.

In addition to the linguistic symptoms, motor speech impairments are another major challenge for stroke survivors, arising from damage to the control of oro-facial, respiratory, and articulatory muscles. These impairments manifest as hypophonia (reduced volume), a strained or hoarse voice, breathy speech with shortened phrase outputs, and gasping between utterances^9^. Additionally, stroke can lead to dysarthria (slurred speech) and apraxia of speech (disruptions in motor planning and sequencing of sounds)^10^, further complicating verbal communication.

Post-stroke recovery is frequently complicated by co-occurring cognitive impairments, including deficits in attention, memory, and executive functioning, which interact with aphasia and motor speech disorders, negatively affecting communication outcomes^11–15^. These combined impairments limit social participation and quality of life, underscoring the need for comprehensive approaches to post-stroke communication assessment and rehabilitation^16^.

Considering the intricate and diverse nature of post-stroke language impairments, it is no surprise that diagnosing and treating post-stroke aphasia remains a challenge. With respect to rehabilitation, the evidence supports early^17^ and intensive^18^ speech therapy, which is difficult to administer in most healthcare settings. While meeting these intensive therapy requirements poses significant challenges for healthcare providers and has broad social and public health implications^19^, technological innovations offer promising solutions.

ASR systems trained on comprehensive pathological speech databases could automate the extraction of clinically relevant speech markers directly from conversational samples, bypassing the need for standardised task designs that require trained administration and are rarely replicated consistently across clinical sites. Automated pipelines can operate on any recorded speech sample, meaning that assessments need not wait for a scheduled clinic visit or a complete neuropsychological battery: a short telephone or telehealth interaction could yield quantitative fluency indices comparable to those obtained in controlled settings. Unlike clinician-administered assessments, which incur costs in specialist time and infrastructure for every evaluation, automated systems have minimal cost once deployed.

Despite recent advances in ASR applications for healthy speech, their performance remains inadequate for clinical applications in pathological speech, with word error rates (WER) ranging from 21% to 45%^20–22^ compared to 9%–13% on healthy speech^23^. The primary obstacle is the lack of large and annotated databases that capture the diverse and complex impairment patterns alluded to earlier^24–26^. Significant progress has been made in developing pathological speech databases, contributing to both linguistic research and ASR advancements. Nevertheless, important gaps remain. AphasiaBank^27^, one of the pioneers in the field and part of the larger TalkBank database, contains data from over 400 people with all-cause aphasia and 200 control participants, featuring discourse tasks such as picture descriptions, story retelling, and procedural discourse. Studies using AphasiaBank have advanced ASR research through architectures like MT-DNN with BLSTM^28^, attention-based models such as E-Branchformer and Conformer^29^, and Whisper^30^. However, it lacks comprehensive clinical and neuroanatomical data, longitudinal recordings to track recovery, and detailed functional or cognitive outcome measures.

Smaller resources targeting specific motor speech impairments include UA-Speech (N=15)^31^ and TORGO (N=8)^32^. Their limited size and focus on short-form speech (e.g., single words or short phrases) reduce their utility for ASR development, as models may learn recording-environment features rather than impairment-specific characteristics^33^. The SEP-28K dataset^34^ offers longer recordings of dysfluent speech with stuttering labels but no word-level transcriptions, restricting its suitability for supervised ASR.

Collectively, existing resources remain inadequate for training ASR models tailored to aphasic speech, due to limited sample sizes, inconsistent task designs, missing clinical and neuroanatomical data, and the absence of longitudinal recordings. These constraints hinder the development of robust models capable of capturing the complexity and variability of aphasic speech.

To address these gaps, an ideal pathological speech database would combine a large and diverse speaker pool with both long-form and short-form speech samples, capturing the full range of impairment types. It should include standardised, high-quality annotations of phonetic distortions, semantic errors, and dysfluencies to support robust ASR development and evaluation. The addition of detailed clinical metadata, neuroanatomical information, and longitudinal recordings would further enable analysis of lesion-language relationships and recovery trajectories over time.

Here, we introduce SONIVA (Speech recOgNItion Validation in Aphasia), a large-scale database developed with the aim of addressing several of these limitations. SONIVA comprises  ≈ 1000 speech samples from stroke survivors, featuring high-quality recordings, linguistic annotations created by trained raters, and paired clinical data. Beyond database construction, we show how speech foundation model fine-tuned on a subset of SONIVA can extract clinically meaningful linguistic features and how acoustic features from SONIVA can be leveraged for disease classification. The SONIVA corpus has been employed in a range of studies: to improve the ASR performance of fine-tuned models^20,35^; to assess their cross-pathology generalization^21^; to learn multimodal latent representations for classification and prediction of stroke outcomes^36^; to extract physiologically informed features derived from glottal signals^37,38^; to infer stroke lesion location and related structural brain features from speech^39,40^ and to conduct automated transcription analysis of single-word naming tasks^41^.

## Methods

The SONIVA database is composed of speech from stroke survivors and control participants. The database includes speech recordings of two long-form picture description tasks and three short-form single-word tasks (naming, reading, and repetition). The short-form tasks originate from participants in the Imperial Comprehensive Cognitive Assessment in Cerebrovascular Disease (IC3) study^42,43^, whilst both IC3 and Predicting Language Outcome and Recovery after Stroke (PLORAS)^44^ study participants contributed to the long-form picture description tasks. Both studies received ethical approval from the UK Health Research Authority (see section below on ethics statement).

IC3 speech data were collected from consecutive patients with clinically confirmed acute stroke, recruited from Imperial College Healthcare NHS Trust hospitals in England. Patients completed cognition and language tests using the IC3 online assessment battery^42,43^ immediately after stroke and at 3, 6, and 12 months post-stroke. Details of all tasks, including language tasks, have been described elsewhere^42^. The assessment typically lasted 90-120 minutes, with duration varying based on impairment severity, with an unlimited number of breaks given to patients where it was clinically indicated. Additional comprehensive clinical data including Montreal Cognitive Assessment (MoCA)^45^, functional outcomes with the Modified Rankin Scale(mRS)^46^ and the stroke severity with the National Institutes of Health Stroke Scale (NIHSS)^47^, as well as neuroimaging MRI scanning, all captured longitudinally after stroke, were acquired as detailed in the study protocol^42,43^. Speech and cognitive data were also collected from 6,364 UK adults aged over 40 without neurological conditions, serving as a control sample via the online IC3 assessment tool. Although this data has been previously published^48^, the recordings lack manual transcriptions across speech tasks and are therefore not included in Table 2.

PLORAS speech data was collected from stroke survivors recruited across multiple UK sites via the NIHR Clinical Research Network. The speech data were supplemented with demographic details (age, gender, handedness, and time post-stroke), manual scoring of spontaneous speech, and gross anatomical information on lesion location^44^.

SONIVA integrates language assessment protocols from both PLORAS and IC3, each employing distinct methodological approaches to language evaluation. Among the 22 tasks in IC3’s assessment battery, six focus on language, with four specifically evaluating spoken output (Table 1). Both IC3 and PLORAS incorporate picture description tasks from the Comprehensive Aphasia Test (CAT)^49^, with IC3 additionally using a custom-designed beach scene. These picture description tasks elicit complete utterances (e.g., “there is a cat”) rather than single-word responses (“cat”), enabling assessment of grammatical structures and descriptive capabilities. Additionally, IC3 uniquely includes single-word tasks to evaluate spoken fluency, self-correction, and phonological processing, integrating semantic assessment within the naming components.

**Table 1:** IC3 Language Assessment Tasks.

Task	Purpose	Method	Trials
Picture Description	Assess spontaneous speech production and fluency	Describe two black-and-white line drawings in complete sentences	2 images, 90 seconds each
Naming	Assess semantic object recognition and verbal output	Name line drawings from related to item from Boston Naming Test	30 items
Reading	Evaluate visual orthographic analysis and verbal output	Read aloud written words on screen	11 items (including 3 non-words)
Repetition	Test auditory phonological analysis and verbal output	Repeat spoken words	20 items (from 40-word pool)

### Speech annotation methodology

The annotation process involved two distinct protocols summarised in Table 1, divided into picture description and single-word tasks (Naming, Reading and Repetition):

#### Picture description annotation

Audio and video recordings were manually transcribed using the Computerised Language ANalysis (CLAN) program^50^, following the Codes for the Human Analysis of Transcripts (CHAT) format^51^. Transcription was conducted by researchers trained in speech and language therapy. The annotation captured five key dimensions:

##### Broad transcription

Each participant’s utterance (“PAR”) was transcribed orthographically with a corresponding IPA (International Phonetic Alphabet) line (“%pho”), capturing both word-level and phonetic-level information. This feature distinguishes SONIVA from many existing pathological speech databases, which do not include phonetic-level annotations. Demographic details such as age, sex, and clinical voice quality (GRBAS score50) were also recorded. Markers agreed to omit diacritics except in cases of severe speech impairment, where velarized/dark ‘’ was used when necessary. Voiceless, labialized, and nasalized diacritics were also applied in such cases. Additionally, the ‘e’ vowel was transcribed for words such as /bed/, rather than using the ‘’ vowel. Regional accent classifications were noted during the transcription by the transcriber. This was determined through a combined approach of speaker nationality data and acoustic-phonetic analyses performed by trained speech therapists.

##### Dysfluencies

Dysfluencies are widely known to be common in stroke survivors. For consistency, transcribers followed the CHAT conventions when denoting pauses. Pauses shorter than 2.0 seconds were coded as “(.)”. For pauses between 2.01 and 5.00 seconds, the code “(..)” was used. Pauses lasting between 5.01 and 10.00 seconds were transcribed as “(...)”. When the pause exceeded 10.00 seconds, the exact duration was specified, e.g., “(12.0)”.

##### Local events

Non-verbal communication, including gestures, mimes, and emotional states, was annotated. For example, a participant gesturing to a cat or miming a cat catching a fish was documented. Emotional states during spontaneous speech, such as frustration, anger, and laughter, were also captured in annotations.

##### Error coding

Transcribers marked four types of language errors: (a) semantic [* s], (b) phonological [* p], (c) morphological [* m], and (d) neologism [* n]. A language error category was further specified by detailing the code if the intention of the speaker was clear e.g., [* s:r] for a semantic error where a related word was used; [* p:w] for a phonological error where a real-word was used; [* p:n] phonological non-word substitution, among other specific language errors^53^.

##### Additional analysis

**Fig. 1:**
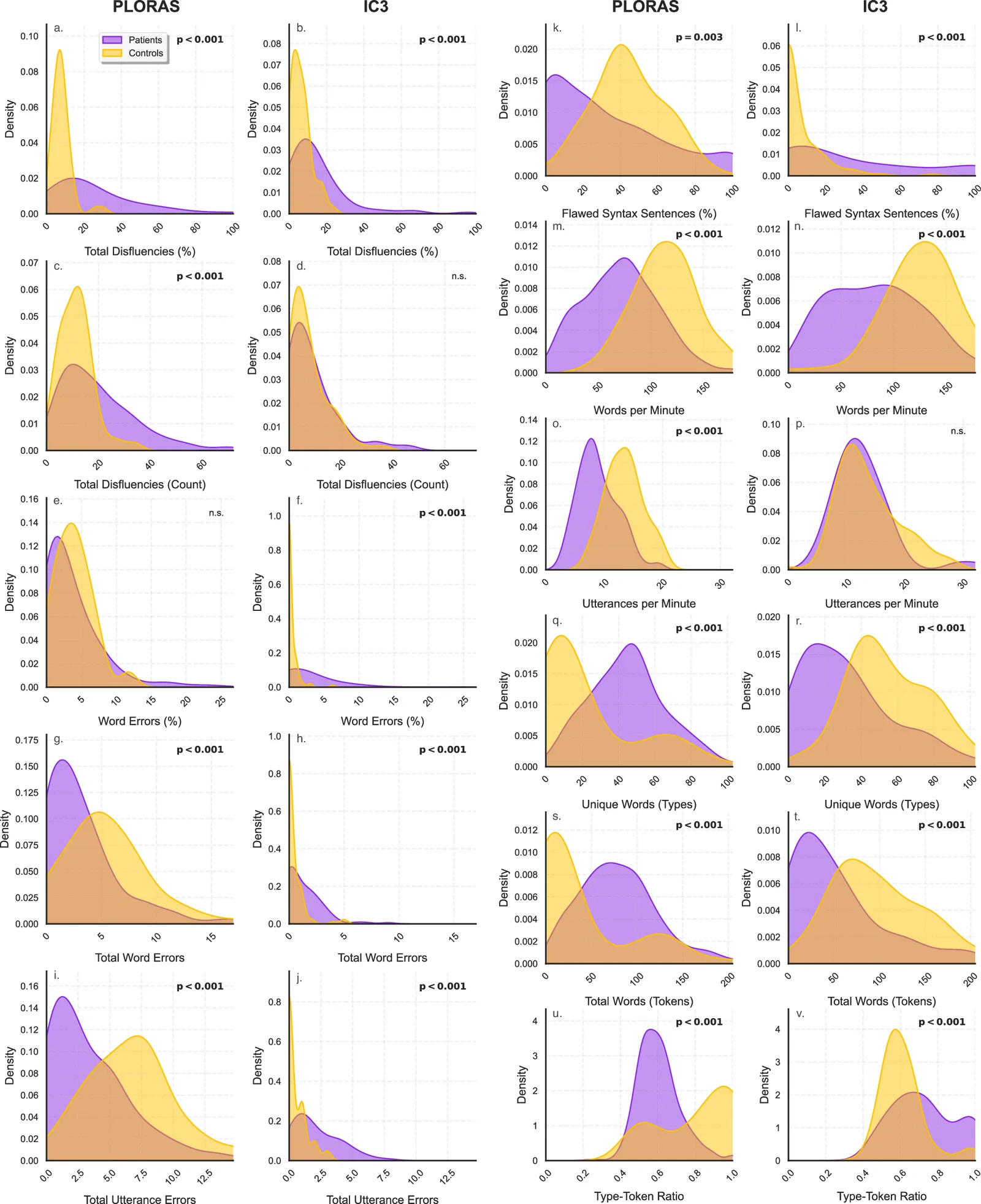
Density plots of CLAN-extracted linguistic features from patient and control transcriptions in the SONIVA database. Data are shown separately for the IC3 (Patients *N* = 42, Controls *N* = 52) and PLORAS (Patients *N* = 529, Controls *N* = 51) picture description protocols. Purple and yellow represent patients and controls, respectively. Subplots are labeled (a)-(v), with each pair of vertically arranged plots representing a single linguistic feature across PLORAS (left) and IC3 (right). P-values  < 0.001 are Bonferroni corrected; “n.s.” indicates non-significant differences.

CLAN commands generated morphological (%mor) and grammatical (%gra) tiers for detailed quantitative linguistic analysis. Profiling commands from CLAN enabled the analysis and quantification of specific language measures from the picture description task, for a total of 11 measures. Measures investigated included the total percentage and count of disfluencies (FLUCALC), total word errors and percentage of word errors (EVAL), total utterance errors (EVAL), percentage of flawed syntax sentences (C-NNLA), words per minute and utterances per minute (Timedur), number of unique words (types), total words (tokens), and type-token ratio (FREQ TTR). The distribution of such measures are reported in Fig. 1.

Transcribers initially underwent group training using a shared set of 10 patient files. Afterwards, six individual transcribers independently annotated a different set of 10 patient files. A two-way mixed-effects intra-class correlation (ICC) analysis was conducted on 15 linguistic measures derived from these 10 independently transcribed recordings to assess inter-rater reliability. These linguistic measures included both error coding variables (4 features) and the additional linguistic analysis metrics (11 features). The ICC analysis aimed to evaluate consistency across transcribers, ensuring that the independently generated annotations were reliable and comparable. Subsequently the individual transcribers proceeded to the transcription of the entire dataset.

The intra-class correlation analysis revealed a high level of inter-rater reliability for the majority of linguistic measures, with most of those achieving the ‘excellent’ criterion (ICC  > 0.90). However, five measures did not reach this highest category. Three measures were rated as ‘good’ (ICC between 0.75 and 0.89): neologism frequency (FREQ_neologism; ICC = 0.860), total word errors (EVAL_word_error_total; ICC = 0.767), and word error percentage (EVAL_word_error_percentage; ICC = 0.724). One measure, morphological errors (FREQ_morphology; ICC = 0.661), was rated as ‘moderate/acceptable’ (ICC between 0.50 and 0.74). Lastly, phonological errors (FREQ_phonology; ICC = 0.311) was rated as ‘poor’ (ICC  < 0.5). For completeness, inter-rater reliability was also evaluated for the GRABS scoring framework, with an overall GRBAS total score of good agreement across annotators (ICC = 0.824).

#### Single-Word task annotation

For IC3, single-word tasks (naming, reading, and repetition) were annotated at the trial level. Each trial was scored on a 0, 1, or 2 scale for each measure - semantic (naming task only), dysfluency, self-correction, and phonology. Higher scores indicated better performance. For patient data, speech was transcribed verbatim as ground truth, including fillers such as “er” and non-informative carrying phrases such as “I don’t know, maybe it’s a...”. The timestamp for all participants’ responses was recorded from the onset time of each target word using Audacity editing software (v. 2.7.3)^54^. Technical errors, background noises, and participant skips due to speech impediments were categorised via specifically agreed-upon codes created by trained transcribers.

### Inclusion & ethics statement

All data collection protocols received ethical approval from the UK Health Research Authority (IC3: IRAS 299333, registered on ClinicalTrials.gov ‘NCT05885295’; PLORAS: IRAS 133939, REC reference 13/LO/1515), and informed consent was obtained from all participants. In addition, the ethical boards granted a consent waiver specifically for the sharing of anonymised transcriptions, enabling public distribution of text data for research purposes. Audio recordings remain classified as personally identifiable information and are subject to additional institutional review and data access agreements prior to any external sharing. The SONIVA database reflects a diverse clinical population, including a broad range of age, gender, regional accents, and neurological severity, supporting inclusive research and model generalizability. No personally identifiable information is shared, and all data handling complies with institutional and GDPR standards.

## Data Record

Speech data from  ≈ 1000 patients with stroke have been collected. More than 100 participants have longitudinal recordings that consist of repeated speech assessments collected at multiple time points after stroke, capturing changes in speech and language over time. The transcribed component of SONIVA database has 26,344 files to date, comprising recordings from 618 stroke survivors and 189 non-stroke control group (Table 2). Picture description tasks contribute to 915 transcriptions, while single-word tasks account for 25,429 transcriptions, providing a diverse linguistic and articulatory representation. Speech recordings from an additional 155 stroke survivors, available but not yet fully transcribed, are not listed in this table. Figure 1 shows the distribution of linguistic features extracted from CLAN for those manually transcribed to date.

**Table 2:** Overview of the annotated SONIVA database composition.

Task Type	Dataset	Controls (files)	Patients (files)	Total Transcriptions
Picture Description	IC3^†^	52 (CAT=65; Beach=66)	42 (CAT=57; Beach=57)	245
	PLORAS	51 (52)	529 (618)	670
	***Total***	***103***	***571***	***915***
SWT-Naming	IC3	138 (8,371)	89 (4,200)	12,571
SWT-Reading	IC3	138 (3,038)	88 (1,518)	4,556
SWT-Repetition	IC3	138 (5,582)	88 (2,720)	8,302
	***Total***	***138***	***89***	***25,429***
	**GRAND TOTAL**^‡^	**189**^†^	**618**^†^	**26,344**

^†^All IC3 picture description participants also completed the Single Word Tasks (SWT). ^‡^Grand total accounts for overlap between IC3 picture description and single word participants.

**Fig. 2:**
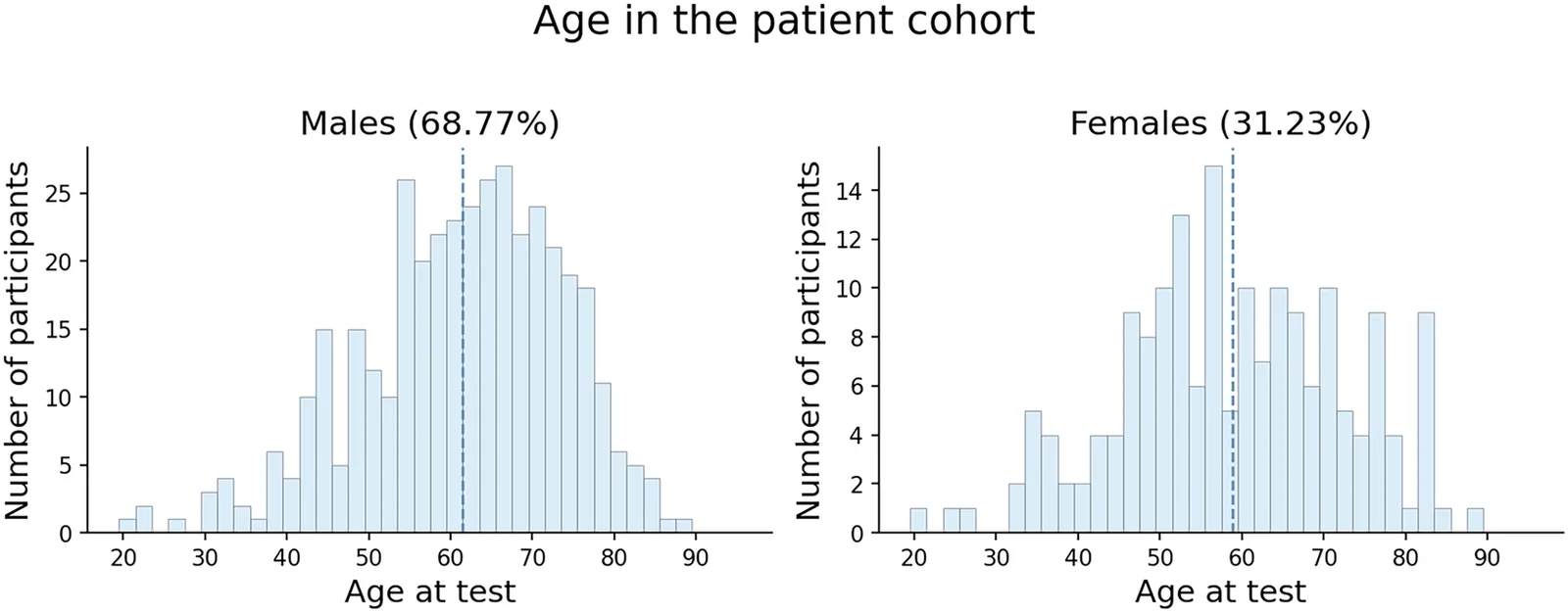
Distribution of age and gender across stroke survivors in the SONIVA database as shown in Table 3.

The age and gender demographics of SONIVA participants align with patterns typically observed in stroke research cohorts (Fig. 2). Among stroke survivors, who make up the majority of the dataset, males account for 68.77% of participants, with an average age of 61.38 years, while females average 58.88 years. This gender imbalance is consistent with global stroke incidence trends, particularly in younger age groups where males are more frequently affected^55^. The control cohort shows a more balanced profile, with males representing 45.63% of participants, and mean ages of 62.05 years for males and 57.61 years for females (overall mean: 59.64  ± 11.48 years). When considering the full SONIVA dataset, including both stroke survivors and controls, males constitute 65.23% of participants, with an overall mean age of 60.50  ± 12.75 years (mean: 61.46 years for males; 58.58 years for females).

**Fig. 3:**
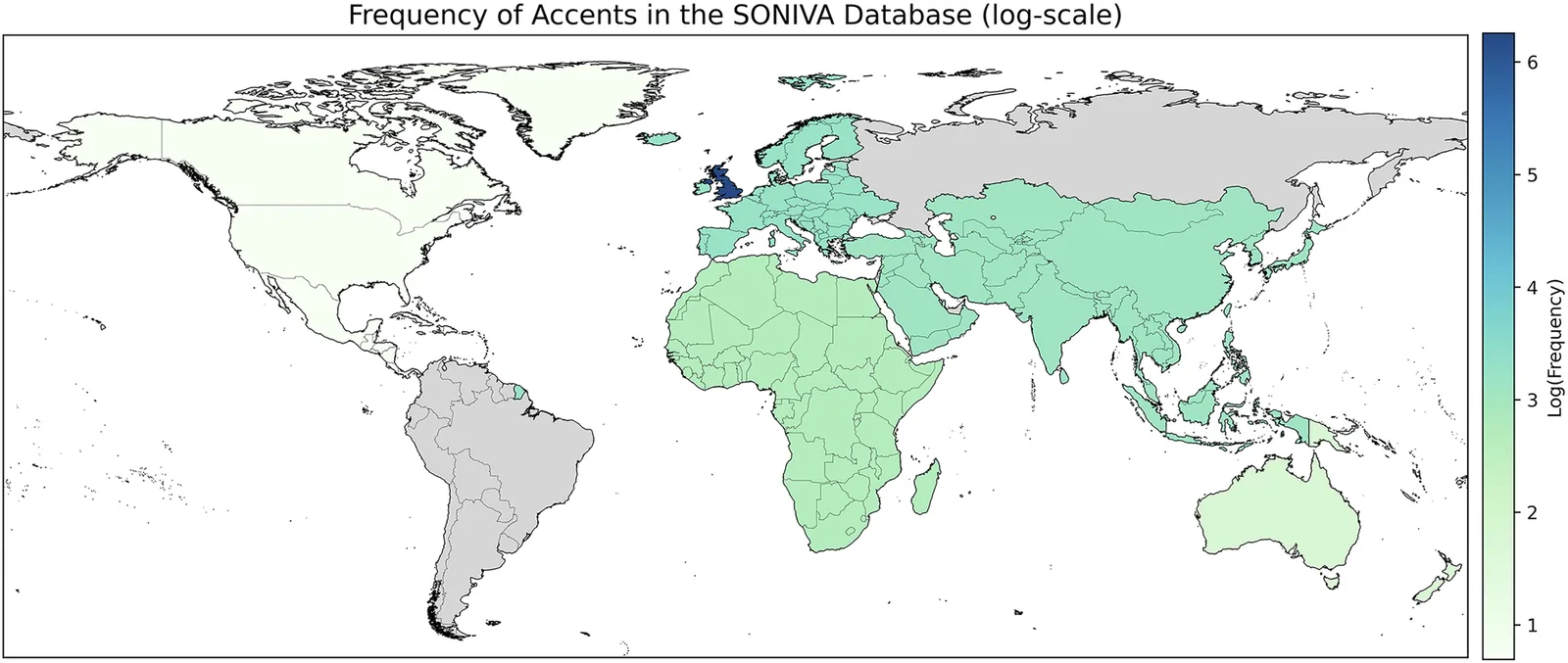
World accent distribution across SONIVA database, including both patients and healthy controls, as shown in Table 3.

SONIVA includes tasks conducted in British English. However, given the clinical nature of the database derived from a metropolitan population, it naturally captures a diverse range of accents, reflecting its broad geographical representation (Fig. 3). This is important to capture, as accents and regional dialects can significantly affect the generalisability of language and acoustic models^56^. The majority of speakers in SONIVA (77.45%) have UK-based regional accents, with the remainder representing a broad geographical range: European (3.71%), Asian (3.26%), Central American (2.67%), African (2.23%), Oceanian (0.74%), and North American (0.30%). A further 9.20% of speakers have undetermined or mixed accents, and 0.45% are classified as non-verbal.

**Fig. 4:**
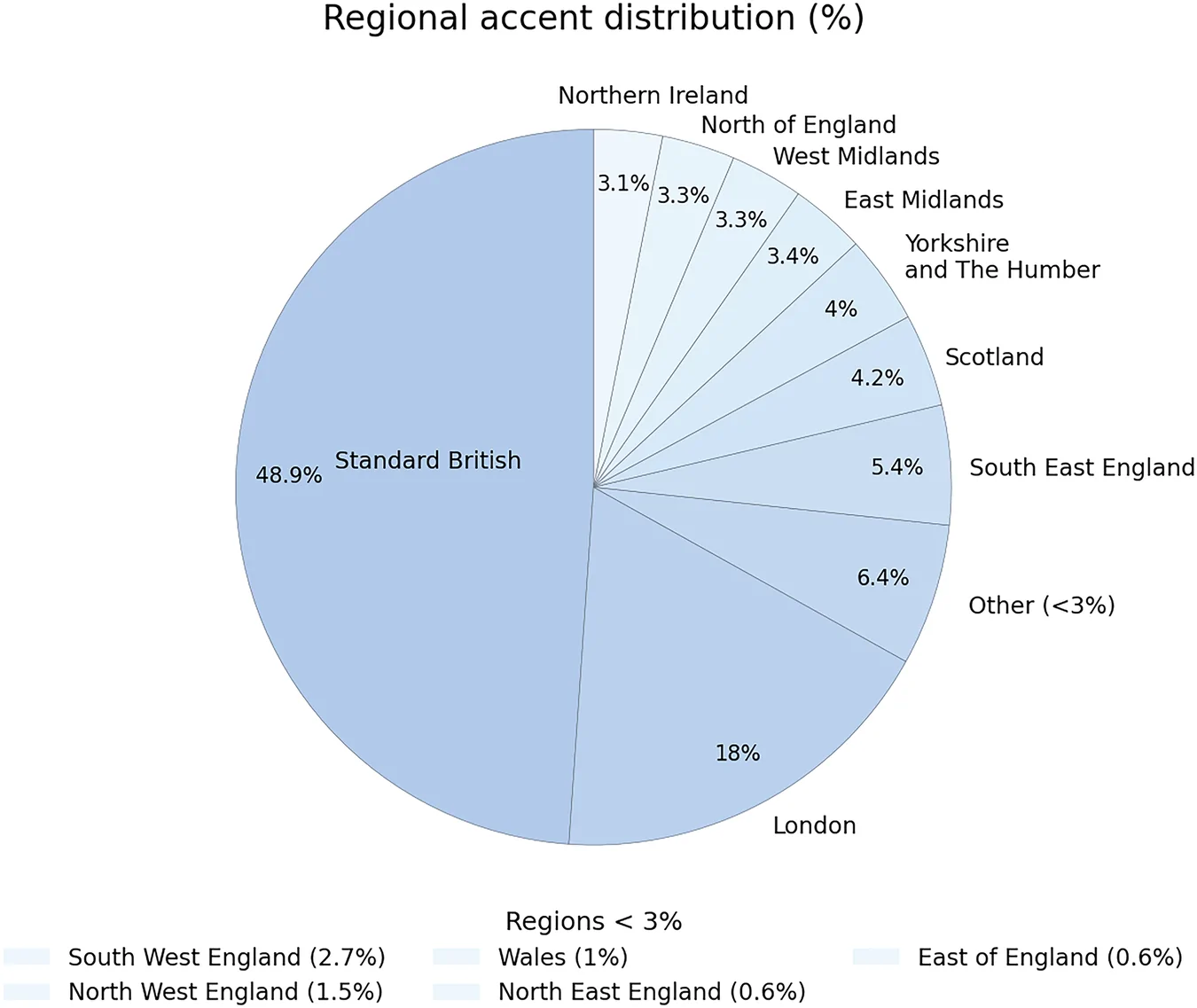
UK accent distribution across the whole SONIVA database as shown in Table 3.

Among UK-based speakers, Fig. 4 shows that 48.9% of participants exhibit “Standard British” accents, a category encompassing neutral English pronunciations without strong regional affiliation. London-area accents account for 18.0% of the cohort. Other prominent regional accents include South East England (5.4%), Scotland (4.2%), Yorkshire and The Humber (4.0%), East Midlands (3.4%), West Midlands (3.3%), and North of England (3.3%), the latter capturing northern inflections that do not align with a single specific region. Smaller proportions are observed for Northern Ireland (3.1%), South West England (2.7%), North West England (1.5%), Wales (1.0%), North East England (0.6%), and East of England (0.6%), highlighting the broad phonetic and geographic diversity represented within the dataset.

**Fig. 5:**
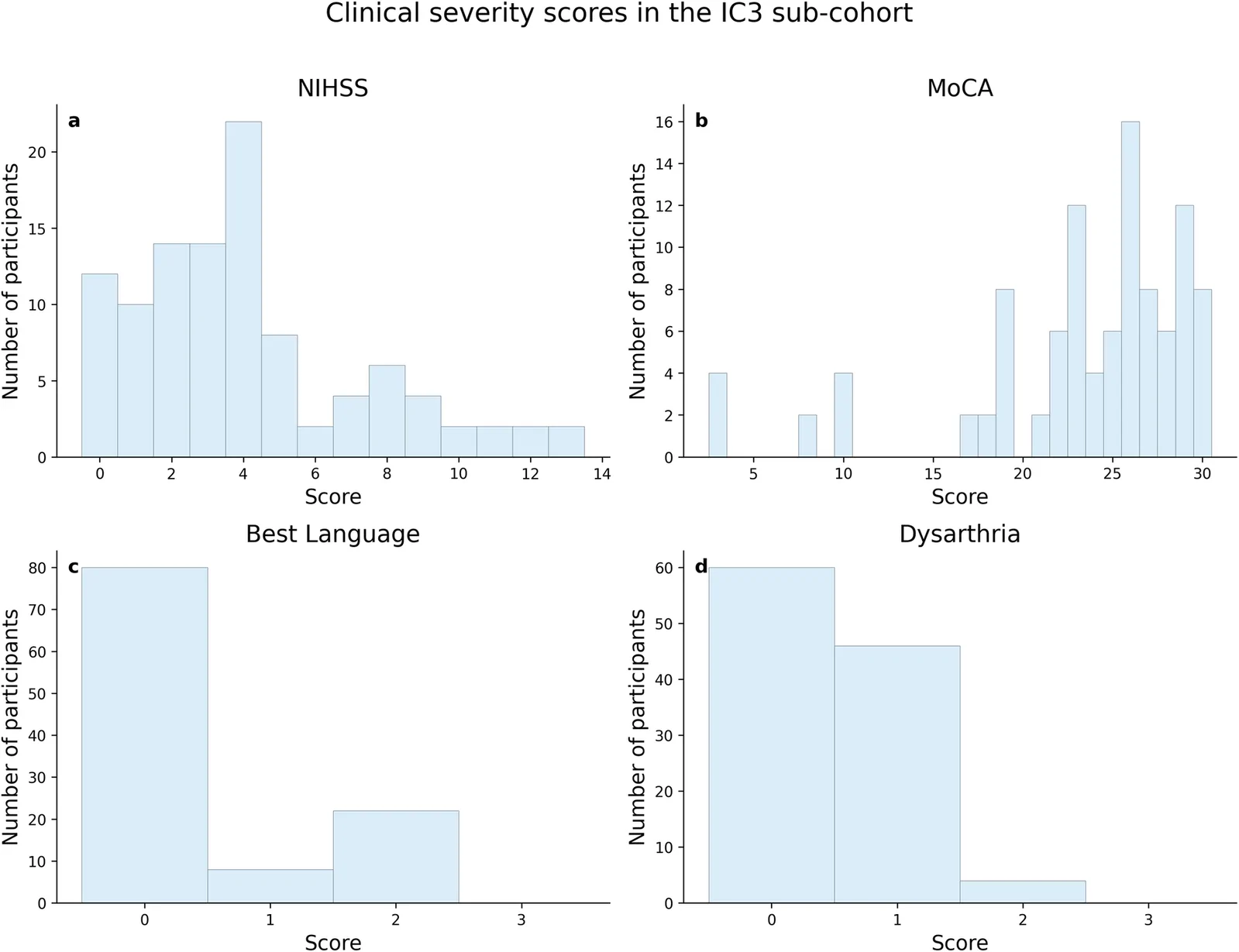
Distribution of clinical scores and severity for the patients in the IC3 cohort. **(a)** NIH Stroke Scale (NIHSS) total severity distribution. The stroke severity levels are defined as ≤5 (minor), 6–10 (mild), 11–15 (moderate), 16–20 (severe), and  > 20 (very severe)^67^. **(b)** Montreal Cognitive Assessment (MoCA) score distribution, where scores  < 26 indicates cognitive impairment^45^. **(c)** NIHSS item 9 (Best Language) score distribution. Scores are defined as 0=normal language, 1=mild-moderate aphasia, 2=severe aphasia, and 3=global aphasia (no usable speech or comprehension)^47^. **(d)** NIHSS item 10 (Dysarthria) score distribution. Scores are defined as 0 = normal articulation, 1 = mild–moderate dysarthria (slurred but intelligible speech), and 2 = severe dysarthria (speech difficult to understand or unintelligible); scores  > 2 indicate mute/anarthric speech^47^.

**Fig. 6:**
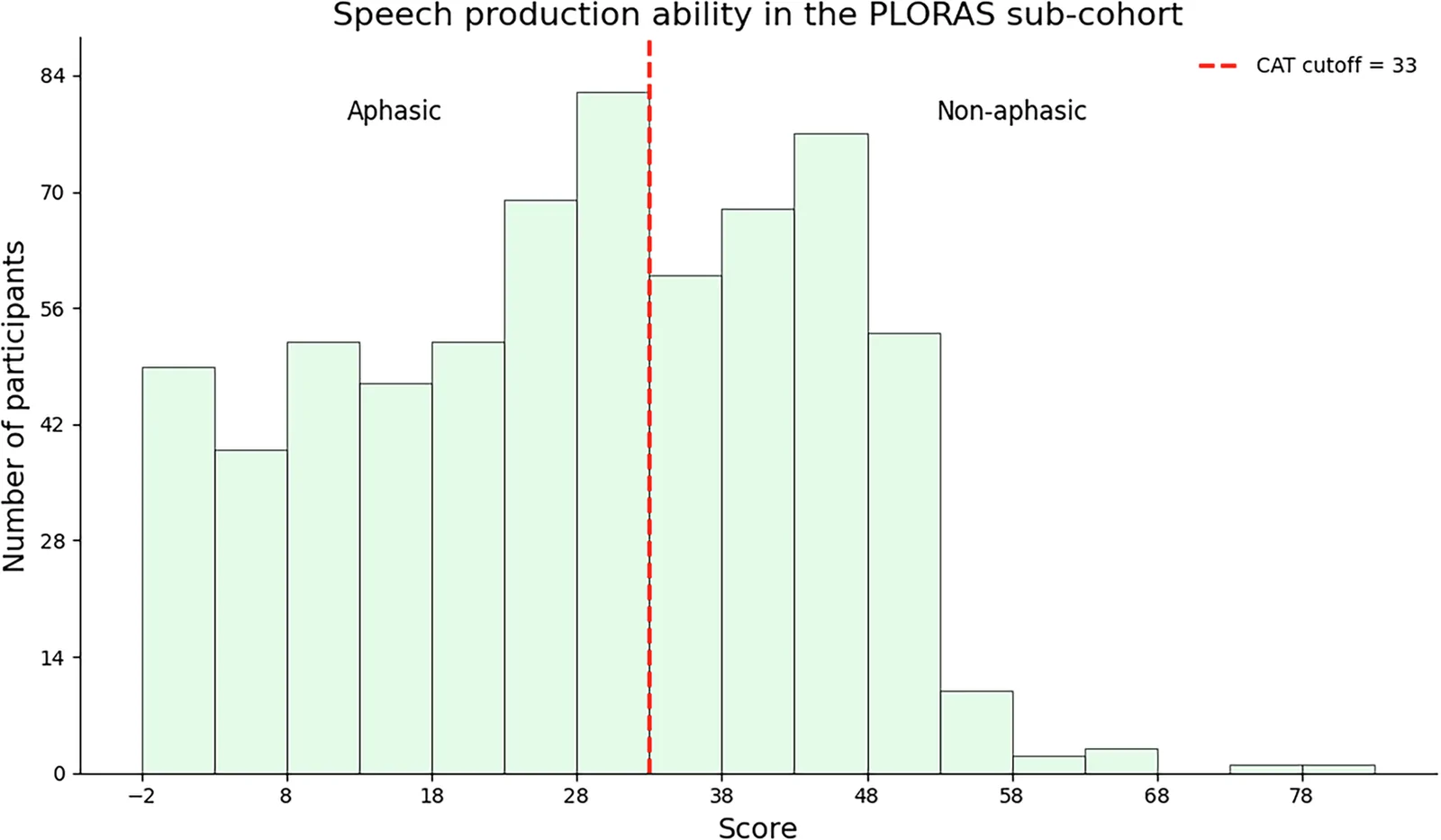
Distribution of speech production ability in the PLORAS sub-cohort. Speech production ability was measured using the total ‘CAT answer scores’, the total score for the Comprehensive Aphasia Test battery picture description task. The red dotted line indicates the clinical cutoff for aphasia as defined in the Comprehensive Aphasia Test^49^.This language-based measure reflects performance in connected speech production but does not capture speech motor impairments such as dysarthria or more subtle aphasic errors.

SONIVA includes patients spanning a wide range of stroke and aphasia severity levels, achieved through the combination of the IC3 and PLORAS cohorts. Clinical severity was assessed differently across the two contributing cohorts. In the IC3 cohort, stroke severity was evaluated using the National Institutes of Health Stroke Scale (NIHSS)^47^, a measure of global neurological impairment following stroke that includes specific subscales for language and dysarthria. The distribution of NIHSS scores and relevant language-related subscales is shown in Fig. 5. In addition, global cognitive status was assessed using the Montreal Cognitive Assessment (MoCA)^45^, which we report for completeness as an auxiliary measure of cognitive functioning. In contrast, clinical severity in the PLORAS cohort was assessed using picture-description scores from the Comprehensive Aphasia Test (CAT)^49^, which capture language-specific functional performance in connected overt speech. The distribution of CAT answer scores for the picture description task is shown in Fig. 6. Voice quality was assessed using the GRBAS scale^52^ to evaluate five perceptual dimensions: Grade (overall severity), Roughness, Breathiness, Asthenia, and Strain, each rated on a 4-point scale from 0 (normal) to 3 (severe). Across the patient cohort of both IC3 and PLORAS, GRBAS ratings were predominantly concentrated in the lower severity range (0-1), indicating generally normal or only mildly altered voice quality. Mean ( ± SD) values were G = 0.30  ± 0.61, R = 0.26  ± 0.56, B = 0.08  ± 0.32, A = 0.13  ± 0.40, and S = 0.05  ± 0.27, corresponding to a total GRBAS score of 0.82  ± 1.78.

**Fig. 7:**
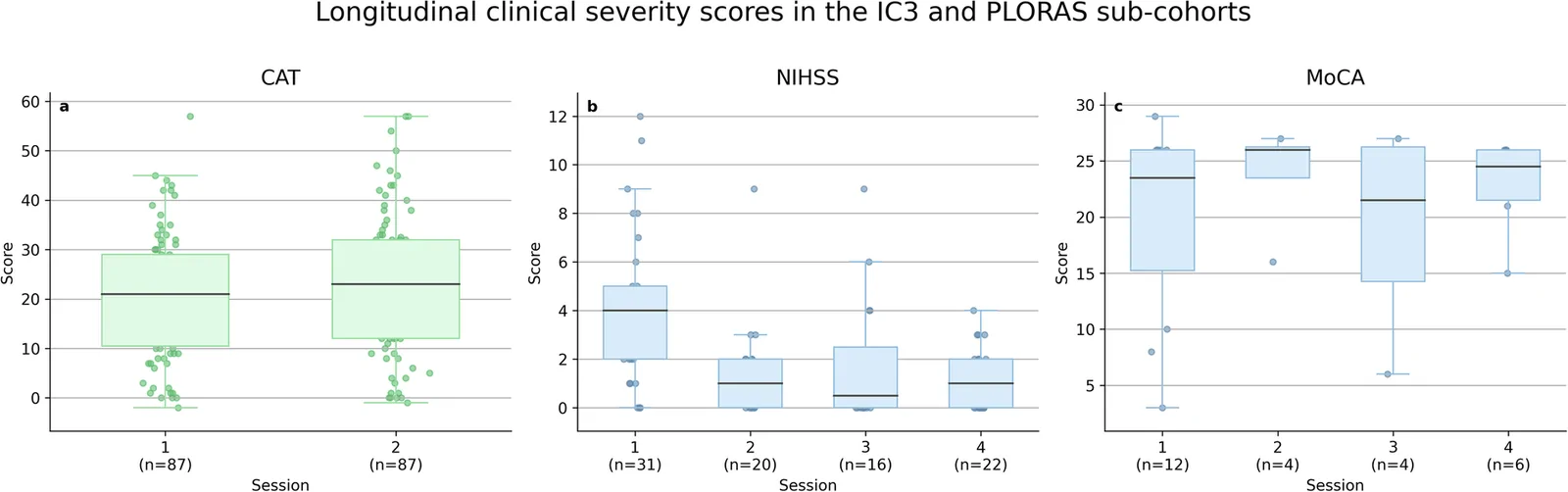
Distribution of clinical severity measures across recording sessions in participants with repeated measures over time. Boxes represent the interquartile range (IQR), the central line indicates the median, and whiskers extend to 1.5 × IQR. Individual data points are overlaid, and sample sizes for each session are reported within each panel. **(a)** PLORAS sub-cohort: Comprehensive Aphasia Test (CAT) total answer scores on the picture description task across two sessions (session 1= baseline; session 2= follow-up). **(b)** NIH Stroke Scale (NIHSS) scores and **(c)** Montreal Cognitive Assessment (MoCA) scores in the IC3 sub-cohort across four sessions (session 1=acute post stroke; sessions 2-4= longitudinal follow-ups over approximately 12 months post-stroke).

To illustrate the longitudinal structure of the SONIVA dataset, we summarise the distribution of clinical severity scores across recording sessions for both sub-cohorts (Fig. 7). CAT Total Answer scores are presented for the PLORAS cohort (Fig. 7a), and NIHSS (Fig. 7b) and MoCA (Fig. 7c) scores for the IC3 cohort. The boxplots capture the distribution of scores at each time point, highlighting variability across participants with repeated measurements.

## Technical Validation

Two experiments were conducted to evaluate SONIVA’s dataset clinical utility. The first leveraged foundation model capabilities for pathological speech analysis by fine-tuning a state-of-the-art automatic speech recognition system to extract linguistic features and compare them with gold-standard human annotations. The second assessed machine learning classifiers trained on acoustic features to distinguish stroke from control speech.

### Foundation model fine-tuning on SONIVA

Whisper^23^, a transformer-based foundation model for automatic speech recognition, was selected for its demonstrated robustness across diverse acoustic conditions and languages. Originally trained on 680,000 hours of multilingual audio data, Whisper is an example of large-scale, generalizable speech recognition systems that can adapt to specialized domains with minimal fine-tuning. This foundation model approach has been explored in the context of pathological speech, where traditional ASR systems face limitations due to acoustic and linguistic variability inherent in neurological conditions. Our previous work has validated Whisper’s effectiveness for post-stroke speech analysis, making it a suitable model for evaluating ASR performance on SONIVA^20,21,35,36^. For this experiment, the medium-sized Whisper model (769M parameters) was employed and fine-tuned on the annotated portion of SONIVA, comprising recordings from 571 stroke survivors performing picture description tasks. The dataset was partitioned into training (70%), validation (18%), and test (12%) sets, stratified by stroke severity and dataset source (i.e. IC3 or PLORAS) to ensure balanced representation across clinical presentations. All parameters were updated without layer freezing, using a per-device batch size of 16, gradient accumulation, and a cosine learning rate schedule (starting at 1 × 10^−5^ with 1,000-step warm-up). Optimisation used AdamW with cross-entropy loss, evaluated by Word Error Rate (WER) every 1,000 steps. Training was capped at 6,000 steps, with the best model selected based on WER. Mixed-precision (fp16) and gradient checkpointing were applied to improve efficiency. Training completed in 8 hours using PyTorch and Hugging Face Transformers on an NVIDIA RTX 6000 GPU.

Following training, the unseen test set was used exclusively for linguistic feature extraction, allowing for an independent assessment of the model’s ability to generalise. Key linguistic features were extracted and correlated with those extracted from human transcriptions. This step allowed assessment of the reliability of the ASR-based analysis, ensuring that the automatically derived features align closely with those obtained from human transcriptions. Using the NLTK library, commonly used for tokenisation, part-of-speech tagging, and stop word identification^57^, 10 linguistic features were extracted and categorised into Lexical Diversity, Syntax, and Dysfluency metrics, capturing key aspects of language complexity and fluency.

(1) Lexical Diversity metrics include *Total Words* (word count, *N*_words_) and *Unique Words*, (distinct tokens, *N*_unique_), from which we also compute the *Type-Token Ratio* ($\frac{{N}_{{\rm{unique}}}}{{N}_{{\rm{words}}}}$), assessing vocabulary variation; and *Lexical Density* ($\frac{{N}_{{\rm{content}}}}{{N}_{{\rm{words}}}}$) quantifies the proportion of content words (*N*_content_).

(2) Syntax structure is evaluated through *Noun-to-Verb Ratio* ($\frac{{N}_{{\rm{nouns}}}}{{N}_{{\rm{verbs}}}}$); The *Function Word Ratio* ($\frac{{N}_{{\rm{function}}}}{{N}_{{\rm{words}}}}$), where function words include prepositions, conjunctions, determiners, pronouns, and auxiliary verbs, indicating syntactic cohesion; and *Part-of-speech Transitions* capturing sentence complexity; *Unique Content Word Variety* ($\frac{{N}_{{\rm{unique}}{\rm{content}}}}{{N}_{{\rm{content}}}}$) measuring the diversity of meaningful words (nouns, verbs, adjectives, and adverbs) capturing the richness of conveyed information.

(3) Dysfluency metrics include *Filler Word Count*, identifying hesitation markers, and *Filler Ratio* ($\frac{{N}_{{\rm{fillers}}}}{{N}_{{\rm{words}}}}$), measuring speech fluidity.

Finally, the linguistic features derived automatically and manually were correlated using a Spearman correlation.

### Acoustic feature analysis

The second experiment aimed to evaluate whether acoustic features extracted from the SONIVA database could reliably distinguish stroke survivors from non-stroke healthy controls.

Acoustic features were extracted using the openSMILE Python library (v. 3.0.1) with the extended Geneva Minimalistic Acoustic Parameter Set (eGeMAPSv02)^58^. This standardised feature set comprises 88 parameters, selected for their relevance in clinical and paralinguistic speech analysis, already widely clinically proven for assessing pathological speech in patients with motor speech sound disorders (rather than the aphasic patient population)^59–63^. The acoustic feature set encompasses prosodic measures (e.g., *F*_0_ stability, jitter, shimmer) to assess pitch and phonatory control, spectral features (e.g., MFCCs, flux) for vocal resonance and articulatory precision, and energy-related metrics (e.g., loudness, harmonics-to-noise ratio, pauses) to evaluate fluency and rhythmic disturbances. All features were included in the classification models.

Audio files were taken from the SONIVA database (N=571 stroke survivors; 103 controls; total N=674 participants). Prior to feature extraction, all recordings were segmented at sentence level so that only participant speech was analyzed, for then merging them before the classification. Using a StratifiedGroupKFold strategy to account for the longitudinal structure of SONIVA, the grouping variable was the participant identifier (ID), ensuring that all segments from a given participant were assigned to the same split and preventing data leakage between training and testing. Stratification was performed using participant label (patient vs. control) and recording source to maintain comparable distributions across splits. Overall, 90% of participants were allocated to the training set and the remaining 10% to the test set (see Table 3).

**Table 3:** Summary of training and test splits for acoustic feature classification, separated by patients and non-stroke controls.

Dataset	Mean Age (SD)	Male (%)	Female (%)	Count
**Train set**				
*Patients*	60.37 (12.86)	352 (68.48%)	162 (31.52%)	514
*Controls*	59.71 (11.50)	43 (46.74%)	49 (53.26%)	92
**Total**	**60.27 (12.65)**	**395 (65.18%)**	**211 (34.82%)**	**606**
**Test set**				
*Patients*	62.46 (13.41)	41 (71.93%)	16 (28.07%)	57
*Controls*	59.14 (11.98)	4 (36.36%)	7 (63.64%)	11
**Total**	**61.92 (13.17)**	**45 (66.18%)**	**23 (33.82%)**	**68**

*Note:* Demographics are calculated per unique patient; counts reflect unique participants.

Three distinct classification models were employed: (1) a Support Vector Machine (SVM) with a radial basis function (RBF) kernel and probability estimates enabled; (2) a Random Forest (RF) classifier with 70 trees, square-root feature subsampling at each split, a maximum tree depth of 20, minimum samples per split of 4, and minimum samples per leaf of 2; and (3) a fully connected Neural Network (NN) for binary classification. The NN comprised an input layer matching the dimensionality of the acoustic feature set, followed by three hidden layers with 64, 32, and 16 units respectively, each using ReLU activation and 15% dropout. The output layer consisted of a single neuron producing raw logits. The model was trained using the AdamW optimizer with a learning rate of 0.001 and weight decay of 10^−4^, alongside a ReduceLROnPlateau learning-rate scheduler and gradient clipping with a maximum norm of 1.0. To address class imbalance, the SVM and RF models employed Synthetic Minority Oversampling Technique (SMOTE)^64^, applied exclusively to the training fold at each cross-validation step to prevent leakage into the validation set. For the NN, SMOTE was not used; instead, a weighted Binary Cross-Entropy loss (BCEWithLogitsLoss) was applied, with the positive-class weight set to the ratio of negative to positive training samples. Additionally, a custom threshold-tuning procedure was applied to the NN: rather than using the default decision boundary of 0.5, the optimal threshold was selected on each validation fold by maximising specificity subject to a minimum sensitivity constraint of 0.90, and the median threshold across folds was applied at test time.

### Experiments result

Fine-tuning the ASR significantly improved its performance, reducing WER from 39.60% to 21.51% on the validation set and from 43.62% to 21.93% on the test set^21^. Nevertheless, residual transcription errors may still affect downstream applications that require detailed linguistic annotation (e.g., speech-error analysis), even if they have minimal impact on broader tasks such as disease classification. Accordingly, the implications of the remaining WER should be evaluated in relation to the specific requirements and sensitivity of each downstream analytical task.

**Fig. 8:**
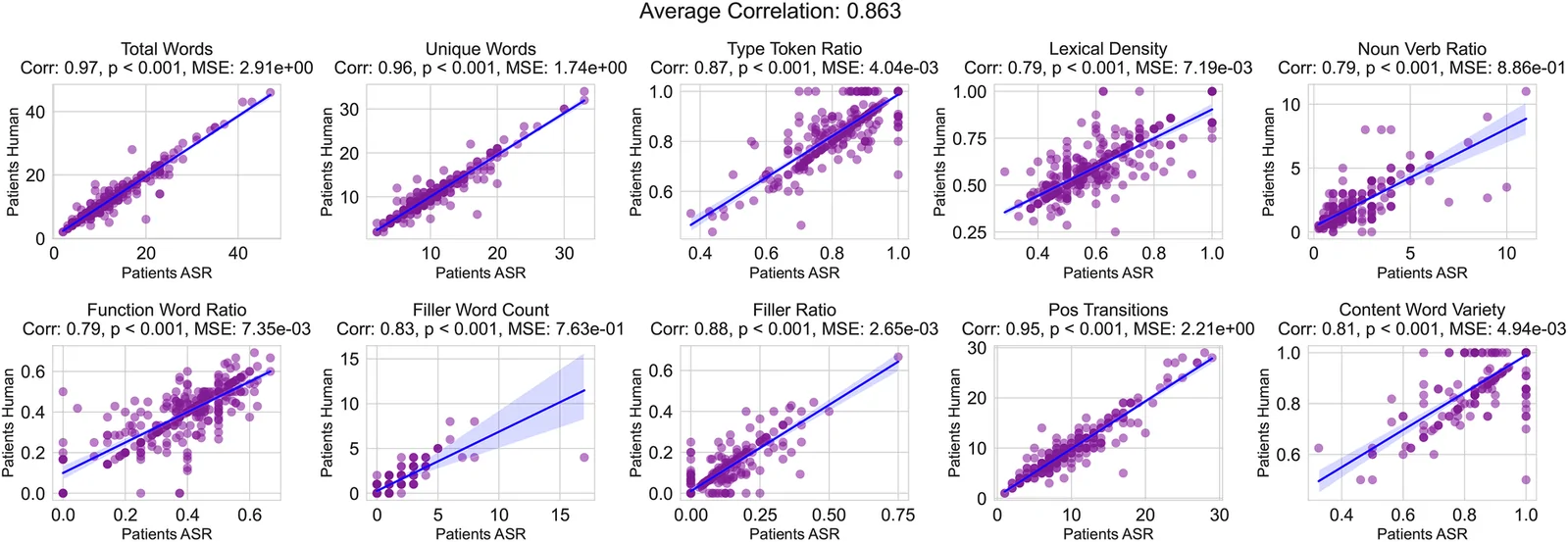
Correlation of linguistic features derived from ASR output and human-labelled speech. For detailed description of the individual linguistic features, see methods. Each point represents a patient (*N* = 571).

The analysis comparing ASR-derived linguistic features with those derived from human transcriptions revealed robust correlations across all measured variables (see results in Fig. 8), yielding an average correlation of 0.863. Basic lexical measures demonstrated strong correlations, with total words, unique words, and type-token ratio showing correlations of 0.97, 0.96, and 0.87, respectively, accompanied by low mean squared errors (MSE: 2.91e+00, 1.74e+00, and 4.04e-03). Lexical density also exhibited notable alignment (Corr: 0.79, MSE: 7.19e-03). Grammatical structure metrics showed high reliability, with function word ratio (Corr: 0.79, MSE: 7.35e-03), part-of-speech transitions (Corr: 0.95, MSE: 2.21e+00), and noun-verb ratio (Corr: 0.79, MSE: 8.86e-01). Measures of disfluency and content analysis similarly displayed strong alignment, with filler word count (Corr: 0.83, MSE: 7.63e-01), filler ratio (Corr: 0.88, MSE: 2.65e-03), and content word variety (Corr: 0.81, MSE: 4.94e-03).

**Fig. 9:**
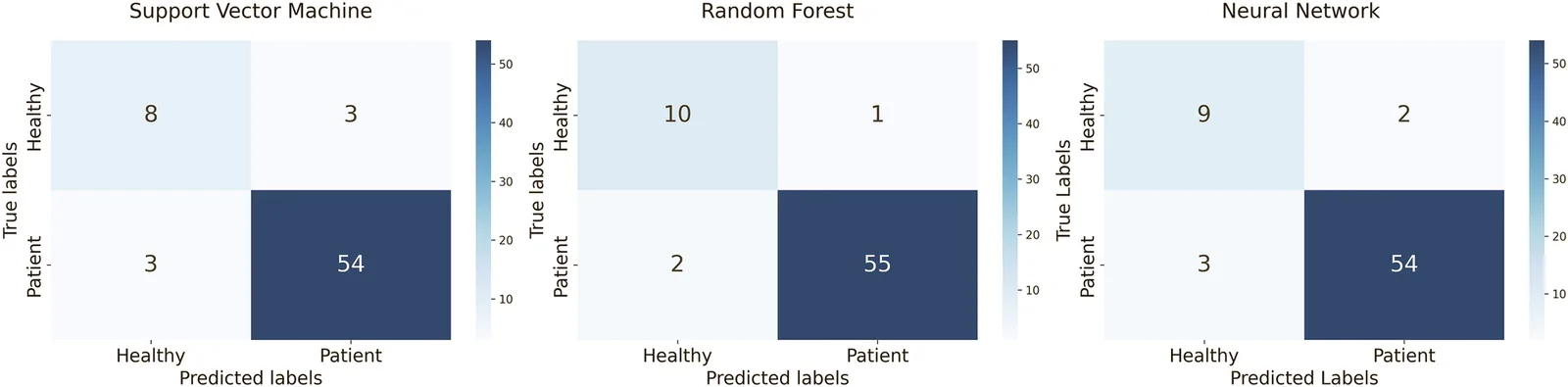
Confusion matrix of the three classification methods used to classify stroke survivors vs. non-stroke healthy controls using acoustic features derived from openSMILE. Colourbar represents absolute numbers.

For the classification performance, Table 4 reports the macro-averaged precision, recall, F1-score, and overall accuracy, while the confusion matrices in Fig. 9 provide the distribution of predictions across classes. Overall, the Random Forest (RF) classifier demonstrated the strongest performance across all metrics, achieving an accuracy of 0.96 and a macro-averaged F1-score of 0.92. The Neural Network (NN) followed with an accuracy of 0.93 (F1 = 0.87), while the Support Vector Machine (SVM) reached 0.91 (F1 = 0.84).

**Table 4:** Performances on the held-out set on the three classification methods using openSMILE acoustic features.

Model	Precision	Recall	F1-score	Accuracy
Support Vector Machine	0.84	0.84	0.84	0.91
Random Forest	**0.91**	**0.94**	**0.92**	**0.96**
Neural Network	0.86	0.88	0.87	0.93

As illustrated in the confusion matrices, the RF model was particularly effective at minimizing errors in the minority class, correctly identifying 10 out of 11 healthy controls with only 1 false positive. It also demonstrated the highest sensitivity for the patient group, correctly classifying 55 out of 57 patients. While the NN and SVM also achieved high accuracy, they exhibited a higher rate of false negatives (3 for both) and false positives (2 and 3, respectively) compared to the RF. These results suggest that while all three architectures can successfully distinguish stroke speech from control recordings using openSMILE features, the RF architecture provides the most robust balance between sensitivity and specificity for this dataset.

Overall, the technical validation procedures presented here were designed to evaluate the internal quality and consistency of the SONIVA dataset. Both experiments, (i) automatic speech recognition fine-tuned on SONIVA transcriptions and (ii) acoustic classification using standardized features, demonstrate the reliability of the annotated data for generating clinically meaningful speech metrics. The high correlations observed between ASR-derived and manually transcribed linguistic features, as well as strong classification performance across models, support the dataset’s integrity and consistency. These results indicate that SONIVA provides a high-quality resource for the development and benchmarking of automated tools in clinical speech analysis, with potential applications in digital health, remote assessment, and personalised therapy.

## Usage Notes

Despite SONIVA’s scale and clinical depth, several limitations should be considered when using the dataset. Firstly, the dataset includes recordings captured in real clinical settings, which may contain background noise, speaker overlap, or other artefacts. These introduce variability that can affect acoustic modelling. Future users may consider applying speaker diarisation, segmentation, or voice activity detection methods to refine analyses and isolate patient speech more precisely.

As described in the Data Records section, although clinical severity was assessed using different instruments across the two contributing cohorts, researchers can harmonise severity levels across cohorts using two complementary strategies. First, a data-driven approach can be adopted by leveraging the provided ground-truth speech transcriptions and the CHAT-derived speech features (e.g. ground truth disfluency measures and/or speech rate in words per minute) to construct cohort-independent severity indicators. Second, severity can be harmonised using a binary classification of impaired and non-impaired based on established clinical cutoffs for the reported severity scores. The relevant cutoff values for the NIHSS and CAT assessments are provided in Figs. 5 and 6.

The experimental validation of SONIVA focused on continuous speech from picture-description tasks, which provide linguistically rich and naturalistic input aligned with the conditions under which contemporary ASR systems are typically trained and evaluated. Isolated single-word productions offer limited temporal and linguistic context for decoding and were therefore not used as the primary basis for ASR validation. In parallel analyses, Whisper performance on SONIVA single-word recordings remained variable, and improvements obtained through fine-tuning were largely task-specific and did not generalise across datasets^41^. Accordingly, the ASR validation reported in this study should be interpreted as applying to continuous speech tasks only. The SONIVA single-word dataset is nevertheless made available to facilitate broader research use following academic request steps described in Data availability section.

Phonological error category demonstrated poor inter-rater reliability (ICC = 0.311), highlighting the complexity of phonological error sub-category classification in post-stroke speech. For example annotators must decide whether an error represents a phonological error with a real-word substitution ([* p:w]), or a non-word substitution ([* p:n]). This difficulty is likely driven by the inherent subjectivity of inferring speaker intent in spontaneous, pathological connected speech. This is a generic problem with pholonogical error sub-categorisation and is not specific to the SONIVA database or the performance of the fine-tuned ASR. Consequently, researchers should exercise caution when performing fine-grained differential diagnoses based solely on subcategory phonological indicators (e.g., p:w vs. p:n).

SONIVA comprises English-language speech from speakers with diverse geographical and accent backgrounds within the UK. While this diversity supports the development of models that account for accent variability within English, the dataset remains limited to a single language. Consequently, models trained on SONIVA will require additional language-specific validation and adaptation before broader cross-linguistic application, in line with the increasing calls for global equity in multilingual and cross-cultural research^65^.

Finally, the current ASR development efforts within SONIVA have focused on the most impaired speakers, for whom human transcription was prioritised. The dataset continues to expand, and future releases may include more automated annotations across milder profiles. These caveats should be taken into account when designing downstream tasks such as classification, longitudinal tracking, or severity prediction.

## Data Availability

All transcriptions from the SONIVA dataset are publicly available. These include orthographic and phonetic (IPA) transcriptions, as well as detailed linguistic error coding (semantic, phonological, morphological errors, and neologisms), all annotated by trained speech and language therapists. Transcriptions from healthy speakers are also included for comparative analyses. In addition, we share anonymised clinical and demographic metadata, which varies depending on the database source (IC3 or PLORAS) that include: • **Demographics:** age, sex, handedness, accent, years of education, and estimated speech/language therapy hours. • **Language and cognitive assessments:** Comprehensive Aphasia Test (CAT) scores (naming, comprehension, syntactic metrics) and related linguistic measures. • **Lesion metrics:** brain lesion volumes segmented by region (e.g., frontal, parietal, subcortical) extracted from neuroimaging. • **Stroke and vascular information:** stroke etiology, lesion site, vascular territories, vessel occlusion, and carotid/vascular imaging modality. • **Clinical scores:** NIHSS (11 subcategories), MOCA, MRS, GRIP, and STAR scores across multiple follow-up sessions. • **Comorbidities and cardiovascular risk factors:** hypertension, high cholesterol, diabetes, atrial fibrillation, previous stroke, smoking history, and other vascular/cognitive risk factors. Speech transcriptions and clinical data can be accessed via Helix Repository^66^ with https://doi.org/10.82186/ra9dv-4az59. This is a fixed, static release of the dataset; additional patient recordings collected in the future will not be added to this version. The fine-tuned speech-to-text model is available at Github repository https://github.com/Clinical-Language-Cognition-Lab/SONIVA_PAPER together with acoustic features extracted from SONIVA audio recordings. To access the data academic institutes can submit a signed copy of the SONIVA Data Usage Agreement available in the gitub repository, and a brief description of the intended research use. Given the sensitivity of the speech audio data, the authors will review individual data access requests and will execute appropriate institutional data sharing agreement depending on the legal status and location of the data requestor. The following conditions must be met: i) The academic requester should have appropriate ethical clearance according to their local regulations, ii) The data needs to be stored in a secure academic research environment, iii) There should be no attempt to re-identify participants, iv) in the unlikely event that a participant is identified through the audio recordings, the authors needs to be informed. Access will be granted via a secure institutional cloud link. This process ensures compliance with UK GDPR and the ethical standards governing sensitive biometric data.

## Code availability

Together with the dataset, the repository https://github.com/Clinical-Language-Cognition-Lab/SONIVA_PAPER contains the acoustic features as well as the fine-tuned Whisper ASR model trained on SONIVA transcripts ready to be used.

## References

[1] Flowers, H. L., Silver, F. L., Fang, J., Rochon, E. & Martino, R. The incidence, co-occurrence & predictors of dysphagia, dysarthria & aphasia after first-ever acute ischemic stroke. *Journal of Communication Disorders* **46**(3), 238–248 (2013). 10.1016/j.jcomdis.2013.04.001 23642855

[2] Ellis, C., Simpson, A. N., Bonilha, H., Mauldin, P. D. & Simpson, K. N. The one-year attributable cost of poststroke aphasia. *Stroke* **43**(5), 1429–1431 (2012). 10.1161/STROKEAHA.111.647339 PMC4507407 22343643

[3] Hill, G. *et al*. Research priorities to improve stroke outcomes. *The Lancet Neurology* **21**(4), 312–313 (2022). 10.1016/S1474-4422(22)00044-8 PMC8926410 35305334

[4] Stefaniak, J. D., Geranmayeh, F. & Lambon Ralph, M. A. The multidimensional nature of aphasia recovery post-stroke. *Brain* **145**(4), 1354–1367 (2022). 10.1093/brain/awab377 PMC9128817 35265968

[5] Damico, J. S., Müller, N. & Ball, M. J. The Handbook of Language and Speech Disorders. Wiley Online Library (2010).

[6] Fridriksson, J. *et al*. Speech entrainment enables patients with Broca’s aphasia to produce fluent speech. *Brain* **135**(12), 3815–3829 (2012). 10.1093/brain/aws301 PMC3525061 23250889

[7] Butler, R. A., Lambon Ralph, M. A. & Woollams, A. M. Capturing multidimensionality in stroke aphasia: mapping principal behavioural components to neural structures. *Brain* **137**(12), 3248–3266 (2014). 10.1093/brain/awu286 PMC4240295 25348632

[8] Fergadiotis, G., Gorman, K. & Bedrick, S. Algorithmic classification of five characteristic types of paraphasias. *American Journal of Speech-Language Pathology* **25**(4), S776–S787 (2016). 10.1044/2016_AJSLP-15-0147 27997952

[9] Charters, E., Coulson, S. & Low, T. Oral incompetence: Changes in speech intelligibility following facial nerve paralysis. *Journal of Plastic, Reconstructive & Aesthetic Surgery* **87**, 472–478 (2023). 10.1016/j.bjps.2022.06.107 37149494

[10] Knollman-Porter, K. Acquired apraxia of speech: a review. *Topics in Stroke Rehabilitation* **15**(5), 484–493 (2008). 10.1310/tsr1505-484 19008207

[11] Lorenz, R. *et al*. A bayesian optimization approach for rapidly mapping residual network function in stroke. *Brain* **144**(7), 2120–2134 (2021). 10.1093/brain/awab109 PMC8370405 33725125

[12] Geranmayeh, F., Leech, R. & Wise, R. J. Network dysfunction predicts speech production after left hemisphere stroke. *Neurology* **86**(14), 1296–1305 (2016). 10.1212/WNL.0000000000002537 PMC4826341 26962070

[13] Geranmayeh, F., Chau, T. W., Wise, R. J., Leech, R. & Hampshire, A. Domain-general subregions of the medial prefrontal cortex contribute to recovery of language after stroke. *Brain* **140**(7), 1947–1958 (2017). 10.1093/brain/awx134 PMC5903407 29177494

[14] Geranmayeh, F., Wise, R. J., Mehta, A. & Leech, R. Overlapping networks engaged during spoken language production and its cognitive control. *Journal of Neuroscience* **34**(26), 8728–8740 (2014). 10.1523/JNEUROSCI.0428-14.2014 PMC4069351 24966373

[15] Geranmayeh, F., Brownsett, S. L. & Wise, R. J. Task-induced brain activity in aphasic stroke patients: what is driving recovery? *Brain* **137**(10), 2632–2648 (2014). 10.1093/brain/awu163 PMC4163030 24974382

[16] Spaccavento, S. *et al*. Quality of life measurement and outcome in aphasia. *Neuropsychiatric Disease and Treatment* **10**, 27 (2014). 10.2147/NDT.S52357 PMC3869916 24368886

[17] Roberts, S. *et al*. Better long-term speech outcomes in stroke survivors who received early clinical speech and language therapy: What’s driving recovery? *Neuropsychological Rehabilitation* **32**(9), 2319–2341 (2022). 10.1080/09602011.2021.1944883 PMC12094264 34210238

[18] Brady, M. C., Kelly, H., Godwin, J., Enderby, P. & Campbell, P. Speech and language therapy for aphasia following stroke. *Cochrane Database of Systematic Reviews* 6 (2016). 10.1002/14651858.CD000425.pub4 PMC8078645 27245310

[19] Le, D., Licata, K. & Provost, E. M. Automatic quantitative analysis of spontaneous aphasic speech. *Speech Communication* **100**, 1–12 (2018).

[20] Sanguedolce, G., Brook, S., Gruia, D. C., Naylor, P. A. & Geranmayeh, F. When Whisper listens to aphasia: Advancing robust post-stroke speech recognition. In *Proceedings of Interspeech* 1995–1999 (2024).

[21] Sanguedolce, G., Gruia, D.-C., Brook, S., Naylor, P. & Geranmayeh, F. Universal speech disorder recognition: towards a foundation model for cross-pathology generalisation. In *Advancements In Medical Foundation Models: Explainability, Robustness, Security & Beyond* (2024).

[22] Hu, S. *et al*. Exploiting cross domain acoustic-to-articulatory inverted features for disordered speech recognition. In *ICASSP 2022–2022 IEEE International Conference on Acoustics, Speech and Signal Processing (ICASSP)* 6747–6751 (2022).

[23] Radford, A. *et al*. Robust speech recognition via large-scale weak supervision. *Proceedings of the 40th International Conference on Machine Learning* **202**, 28492–28518 (2023).

[24] Mahmoud, S. S., Pallaud, R. F., Kumar, A., Faisal, S., Wang, Y. & Fang, Q. A comparative investigation of automatic speech recognition platforms for aphasia assessment batteries. *Sensors* **23**(2), 857 (2023). 10.3390/s23020857 PMC9863375 36679654

[25] Le, D. & Provost, E. M. Improving automatic recognition of aphasic speech with AphasiaBank. In *Interspeech* 2681–2685 (2016).

[26] Fraser, K. C., Rudzicz, F., Graham, N. & Rochon, E. Automatic speech recognition in the diagnosis of primary progressive aphasia. In *Proceedings of the Fourth Workshop on Speech and Language Processing for Assistive Technologies* 47–54 (2013).

[27] MacWhinney, B., Fromm, D., Forbes, M. & Holland, A. AphasiaBank: Methods for studying discourse. *Aphasiology* **25**(11), 1286–1307 (2011). 10.1080/02687038.2011.589893 PMC3424615 22923879

[28] Liu, Y., Qin, Y., Feng, S., Lee, T. & Ching, P. Disordered speech assessment using Kullback–Leibler divergence features with multi-task acoustic modeling. In *2018 11th International Symposium on Chinese Spoken Language Processing (ISCSLP)* 61–65 (2018).

[29] Tang, J. *et al*. A New Benchmark of Aphasia Speech Recognition and Detection Based on E-Branchformer and Multi-task Learning. *Proc. Interspeech* **2023** (2023).

[30] Zusag, M., Wagner, L. & Bloder, T. Careful Whisper – leveraging advances in automatic speech recognition for robust and interpretable aphasia subtype classification. In *Interspeech 2023*3013–3017 (2023).

[31] Kim, H. *et al*. UAspeech 10.21227/f9tc-ab45. (2023).

[32] Rudzicz, F., Namasivayam, A. K. & Wolff, T. The TORGO database of acoustic and articulatory speech from speakers with dysarthria. *Language Resources and Evaluation* **46**, 523–541 (2012).

[33] Schu, G., Janbakhshi, P. & Kodrasi, I. On using the UA-Speech and TORGO databases to validate automatic dysarthric speech classification approaches. In *ICASSP 2023–2023 IEEE International Conference on Acoustics, Speech and Signal Processing (ICASSP)* 1–5 (2023).

[34] Lea, C., Mitra, V., Joshi, A., Kajarekar, S. & Bigham, J. P. SEP-28K: A dataset for stuttering event detection from podcasts with people who stutter. In *ICASSP 2021–2021 IEEE International Conference on Acoustics, Speech and Signal Processing (ICASSP)* 6798–6802 (2021).

[35] Sanguedolce, G., Naylor, P. A. & Geranmayeh, F. Uncovering the potential for a weakly supervised end-to-end model in recognising speech from patient with post-stroke aphasia. In *Proceedings of the 5th Clinical Natural Language Processing Workshop* 182–190 (2023).

[36] Sanguedolce, G., Gruia, D.-C., Naylor, P. & Geranmayeh, F. Latent representation encoding and multimodal biomarkers for post-stroke speech assessment. In *ICLR 2025 Workshop on World Models: Understanding, Modelling and Scaling* (2025).

[37] Sanguedolce, G. *et al*. Speech-based neurological assessment using voice source feature analysis in stroke survivors. In *2025 25th International Conference on Digital Signal Processing (DSP)* 1–5 (2025).

[38] Sanguedolce, G. *et al*. Physiologically-informed feature analysis of acquired speech disorders for stroke assessment. In *Proceedings of Interspeech 2025* 813–817 (2025).

[39] Sanguedolce, G., Parkinson, N. V., Geranmayeh, F. & Naylor, P. A. Attention-based fusion of speech fluency features for stroke lesion mapping. *2026 34th European Signal Processing Conference (EUSIPCO)* (2026).

[40] Sanguedolce, G., Parkinson, N. V., Geranmayeh, F. & Naylor, P. A. Transferable lesion-supervised speech representations for post-stroke modelling. *ICML 2026 Structured Data For Health Workshop* (2026).

[41] Davudova, M. *et al*. Application of Whisper in clinical practice: the post-stroke speech assessment during a naming task. In *The Second Workshop on GenAI for Health: Potential, Trust & Policy Compliance* https://openreview.net/forum?id=1P8VudBhpW. (2025).

[42] Gruia, D. C. *et al*. Online monitoring technology for deep phenotyping of cognitive impairment after stroke. *medRxiv* (2024). 10.1177/10731911251381572 PMC13675564 41147646

[43] Gruia, D.-C. *et al*. IC3 protocol: a longitudinal observational study of cognition after stroke using novel digital health technology. *BMJ Open* **13**(11), e076653 (2023). 10.1136/bmjopen-2023-076653 PMC10679983 38000822

[44] Seghier, M. L. *et al*. The PLORAS database: a data repository for predicting language outcome and recovery after stroke. *NeuroImage* **124**, 1208–1212 (2016). 10.1016/j.neuroimage.2015.03.083 PMC4658335 25882753

[45] Nasreddine, Z. S. *et al*. The Montreal Cognitive Assessment, MoCA: a brief screening tool for mild cognitive impairment. *Journal of the American Geriatrics Society* **53**(4), 695–699 (2005). 10.1111/j.1532-5415.2005.53221.x 15817019

[46] Broderick, J. P., Adeoye, O. & Elm, J. Evolution of the modified Rankin Scale and its use in future stroke trials. *Stroke* **48**(7), 2007–2012 (2017). 10.1161/STROKEAHA.117.017866 PMC5552200 28626052

[47] Kwah, L. K. & Diong, J. National Institutes of Health Stroke Scale (NIHSS). *Journal of Physiotherapy* **60**(1), 61 (2014). 10.1016/j.jphys.2013.12.012 24856948

[48] Gruia, D.-C. *et al*. Development and validation of the IC3: An online remote assessment technology for deep phenotyping and monitoring of cognitive impairment after stroke. *Assessment* 10731911251381572 (2025). 10.1177/10731911251381572 PMC13675564 41147646

[49] Swinburn, K., Porter, G. & Howard, D. Comprehensive Aphasia Test. *APA PsycTests* (2004).

[50] Conti-Ramsden, G. CLAN (Computerized Language Analysis). *Child Language Teaching and Therapy* **12**(3), 345–349 (1996).

[51] MacWhinney, B. The CHILDES Project: Tools for Analyzing Talk, Volume II: The Database. Psychology Press (2014).

[52] Hirano, M. Clinical Examination of Voice. (1981).

[53] MacWhinney, B. Tools for analyzing talk part 2: The CLAN program. *TalkBank.org* 2000 (2017).

[54] Audacity Team Audacity. Free and open-source audio editor and recorder. https://www.audacityteam.org/ (2023).

[55] Appelros, P., Stegmayr, B. & Terént, A. Sex differences in stroke epidemiology: a systematic review. *Stroke* **40**(4), 1082–1090 (2009). 10.1161/STROKEAHA.108.540781 19211488

[56] Yadavalli, A., Mirishkar, G. S. & Vuppala, A. Exploring the effect of dialect mismatched language models in Telugu automatic speech recognition. In *Proceedings of the 2022 Conference of the North American Chapter of the Association for Computational Linguistics: Human Language Technologies: Student Research Workshop* 292–301 (2022).

[57] Bird, S., Klein, E. & Loper, E. Natural Language Processing with Python: Analyzing Text with the Natural Language Toolkit. O’Reilly Media, Inc. (2009).

[58] Eyben, F. *et al*. The Geneva Minimalistic Acoustic Parameter Set (GeMAPS) for voice research and affective computing. *IEEE Transactions on Affective Computing* **7**(2), 190–202 (2015).

[59] Shahin, M., Ahmed, B., Smith, D. V., Duenser, A. & Epps, J. Automatic screening of children with speech sound disorders using paralinguistic features. In *2019 IEEE 29th International Workshop on Machine Learning for Signal Processing (MLSP)* 1–5 (2019).

[60] Barche, P., Gurugubelli, K. & Vuppala, A. K. Towards automatic assessment of voice disorders: A clinical approach. In *INTERSPEECH* 2537–2541 (2020).

[61] Liu, Y. *et al*. Automatic assessment of Parkinson’s disease using speech representations of phonation and articulation. *IEEE/ACM Transactions on Audio, Speech & Language Processing* **31**, 242–255 (2022).

[62] Mawalim, C. O., Titalim, B. A., Okada, S. & Unoki, M. Non-intrusive speech intelligibility prediction using an auditory periphery model with hearing loss. *Applied Acoustics* **214**, 109663 (2023).

[63] Kumar, D., Satija, U. & Kumar, P. Pathological speech and electroglottography signals analysis using invariance scattering network. *Circuits, Systems & Signal Processing* 1–18 (2024).

[64] Chawla, N. V., Bowyer, K. W., Hall, L. O. & Kegelmeyer, W. P. SMOTE: synthetic minority over-sampling technique. *Journal of Artificial Intelligence Research* **16**, 321–357 (2002).

[65] García, A. M., de Leon, J., Tee, B. L., Blasi, D. E. & Gorno-Tempini, M. L. Speech and language markers of neurodegeneration: a call for global equity. *Brain* **146**(12), 4870–4879 (2023). 10.1093/brain/awad253 PMC10690018 37497623

[66] Sanguedolce, G. *et al*. SONIVA Database: Speech Recognition Validation in Aphasia. Dataset 10.82186/ra9dv-4az59 (2026). PMC13623922 42288504

[67] Kogan, E. *et al*. Assessing stroke severity using electronic health record data: a machine learning approach. *BMC Medical Informatics and Decision Making* **20**(1), 8 (2020). 10.1186/s12911-019-1010-x PMC6950922 31914991

